# Antihypertensive medications and risk of colorectal cancer in British Columbia

**DOI:** 10.3389/fphar.2023.1301423

**Published:** 2023-11-07

**Authors:** Jia Qi, Parveen Bhatti, John J. Spinelli, Rachel A. Murphy

**Affiliations:** ^1^ School of Population and Public Health, University of British Columbia, Vancouver, BC, Canada; ^2^ Cancer Control Research, BC Cancer, Vancouver, BC, Canada

**Keywords:** antihypertensive medications, colorectal cancer, risk, safety, cohort

## Abstract

**Introduction:** There is conflicting evidence for the association between antihypertensive medications and colorectal cancer risk, possibly reflecting methodological limitations of previously conducted studies. Here, we aimed to clarify associations between commonly prescribed antihypertensive medication classes and colorectal cancer risk in a large, retrospective, cohort study.

**Methods:** Using linked administrative data between 1996 and 2017 from British Columbia, we identified a cohort of 1,693,297 men and women who were 50 years of age or older, initially cancer-free and nonusers of antihypertensive medications. Medication use was parameterized as ever use, cumulative duration, and cumulative dose. Cox proportional hazard models were used to estimate hazard ratios (HRs) and associated 95% confidence intervals (95% CIs) for associations of time-varying medication use [angiotensin-converting enzyme inhibitors (ACEIs), angiotensin II receptor blockers (ARBs), beta-blockers (BBs), calcium channel blockers (CCBs), and diuretics] with colorectal cancer risk.

**Results:** There were 28,460 incident cases of colorectal cancer identified over the follow-up period (mean = 12.9 years). When medication use was assessed as ever/never, diuretics were associated with increased risk of colorectal cancer (HR 1.08, 95% CI 1.04–1.12). However, no similar association was observed with cumulative duration or cumulative dose of diuretics. No significant associations between the other four classes of medications and colorectal cancer risk were observed.

**Conclusion:** No compelling evidence of associations between antihypertensive medications and colorectal cancer were observed.

## 1 Introduction

Colorectal cancer is the fourth most commonly diagnosed cancer in Canada (Lin et al., 2016). Of those who are diagnosed, about 60% are diagnosed with advanced stage (III or IV) disease (Lin et al., 2016), for which survival is poor (De Oliveira et al., 2009). Although advancements in screening and treatment have improved prognosis (Li, 2018), colorectal cancer is still the second and third leading cause of death from cancer in men and women, respectively (Lin et al., 2016).

Hypertension is one of the most prevalent chronic diseases in Canada affecting approximately 25% of Canadian adults (Padwal et al., 2016). Given the high prevalence of hypertension, antihypertensive drugs are the most commonly prescribed medications in Canada, with over 4 million antihypertensive medication prescriptions written every month (Summers, 2010). Angiotensin-converting enzyme inhibitors (ACEIs), angiotensin II receptor blockers (ARBs), beta-blockers (BBs), calcium channel blockers (CCBs), and diuretics are the most widely used classes of antihypertensive medications (Assimes et al., 2008).

Evidence suggests that the five classes of antihypertensive medications may both inhibit and promote cellular processes involved in carcinogenesis, prompting studies of their possible impact on cancer development, including colorectal cancer (Qi et al., 2022). Multiple studies have evaluated antihypertensive medications in association with colorectal cancer risk, but results have been mixed (Tenenbaum et al., 2001; Boudreau et al., 2008; van der Knaap et al., 2008; Chang et al., 2011; Hallas et al., 2012; Jansen et al., 2012; Deshpande, 2013; Wang et al., 2013; Makar et al., 2014; Chang et al., 2015; Dierssen-Sotos et al., 2017; Mackenzie et al., 2017; Cheung et al., 2020; Cho et al., 2021). A meta-analysis suggested that the five classes of antihypertensive medications are not associated with colorectal cancer risk (Qi et al., 2022). However, the included studies had various methodologic limitations. None of the included studies considered changes in antihypertensive medication use over time (Qi et al., 2022). Hypertensive patients may be prescribed different antihypertensive medications over time (Mann, 2020), or as a result of changes to medical guidance on treatment of hypertension (155). Even for those who use the same medication, the dose of a given medication may need to be adjusted over time to achieve adequate control of blood pressure (Mann, 2020). Furthermore, the bulk of studies only considered ever/never use instead of cumulative durations and doses (Qi et al., 2022). Additionally, cancer in different areas of the colon (i.e., proximal colon, distal colon, and rectum) may have different etiologies (Iacopetta, 2002), but, associations with anti-hypertensive medications and site-specific colorectal cancer risk have been seldom assessed (Tenenbaum et al., 2001; Jansen et al., 2012; Deshpande, 2013; Chang et al., 2015; Mackenzie et al., 2017).

To address these limitations, we examined associations between the five classes of antihypertensive medications and various subclasses with overall and site-specific risks of colorectal cancer using a time-varying analytical approach, in which different measurements of antihypertensive medications were used including ever use, cumulative duration, and cumulative dose.

## 2 Materials and methods

The study was approved by the Research Ethics Boards of the University of British Columbia and BC Cancer (H20-02366).

### 2.1 Study design and data resources

We conducted a retrospective cohort study using administrative data captured from 1 January 1996 to 31 December 2017 for residents of British Columbia (BC), Canada housed at Population Data BC (PopData) (Ark et al., 2019). PopData facilitates access to data from federal and provincial (BC) data sources that are linked to an individuals’ personal health number (PHN) (Ark et al., 2019). Age at cohort entry (index age), sex, the health authority where an individual lived at the time of cohort entry, and income quintiles for neighborhood of residence at the time of cohort entry were ascertained from the Consolidation File. Mortality data were retrieved from the Vital Statistics Deaths file. Date of departure from the province of BC was ascertained from Medical Services Plan (MSP) records. Information about antihypertensive medication use including dispensed medications, date of dispensing, dispensed quantity, and day supply were ascertained from PharmaNet. Cancer diagnoses, diagnosis date, and tumor site were available via linkage with the BC Cancer Registry. BC Cancer Registry data from 1985 to 1995 was used to identify previous and prevalent cancer patients at the time of cohort entry. The BC Cancer Registry captures >95% of cancers within the province from hematology and pathology reports, death certificates, hospital reports, and cancer treatment centers (Ark et al., 2019).

To examine the impact of additional potential confounding factors, data were also drawn from the BC Generations Project (BCGP) which is linkable to PopData via PHNs. The BCGP is a prospective, longitudinal cohort study that began in 2009 with the goal of learning more about how environment, lifestyle and genes contribute to cancer and other chronic diseases (Dhalla et al., 2019). By 2016, 29,850 participants, between the ages of 35–69 years, had been enrolled (Dhalla et al., 2019). Additional possible confounding factors included education (bachelor’s degree or higher, some postsecondary, or high school or less), marital status [married or living with a partner or living without a partner (divorced, separated, or widowed, and single or never married)], household income (>$100,000, $50,000–$100,000, or <$50,000), body mass index (BMI) (normal, underweight, overweight, or obese), vegetable and fruit consumption (≥5 servings per day or <5 servings per day), alcohol consumption (never to moderate or daily), smoking status (ever or never), moderate-to-vigorous physical activity (≥150 min/week or <150 min/week), history of engagement in colorectal cancer screening via use of a fecal occult blood test (ever or never), sigmoidoscopy or colonoscopy (ever or never) and additional possible predictors including ethnicity (white or other), family history of cancer (no or yes), and history of polyp removal (no or yes). Variable categorizations were chosen according to previous studies (Winawer et al., 1990; Mandel et al., 2000; Lin et al., 2014; Clinton et al., 2020; Murphy et al., 2022).

### 2.2 Study population

Participants were drawn from residents in the province of BC with at least five consecutive years of MSP records who were 50 years of age or older between 1996 and 2017 (*n* = 2,201,780). This age range was chosen as 93% of colorectal cancer cases occur in adults aged 50 years or older (Brenner et al., 2020). Any individual with a cancer diagnosis other than non-melanoma skin cancer between 1985 and entry to the cohort were excluded (*n* = 89,788). Individuals who were younger than 50 years of age as of 1 January 1996 could subsequently enter the cohort when they turned 50. Follow-up for each individual was from time of cohort entry to 1) date of any cancer diagnosis other than non-melanoma skin cancer, 2) death, 3) leaving the province of BC, or 4) end of the study period (31 December 2017), whichever occurred first. To minimize the influence of previous use of antihypertensive medications (Assimes et al., 2008), a new-user study design was used whereby individuals who used any antihypertensive medication within the first 2 years of entry into the cohort were excluded (*n* = 418,695).

### 2.3 Exposures

The identification of relevant exposures for this study was informed by the Hypertension Canada Guidelines, Canada’s clinical practice guidelines for management of hypertension (Rabi et al., 2020). This includes initial and second-line therapy as well as additive therapy (i.e., loop diuretics). Use of ACEIs, ARBs, BBs, CCBs, and diuretics were subsequently identified using American Hospital Formulary Service (AHFS) codes (Supplementary Table S1) (Pharmacists and Service, 2002). Subclasses of these medications were identified, including β1 blockers vs. β1/β2 blockers, dihydropyridines vs. non-dihydropyridines, and thiazide vs. loop vs. potassium sparing diuretics (Chen et al., 2017). Medication use was parameterized as: 1) a binary exposure (ever or never use), 2) cumulative duration of medication use (0, >0–2, >2–5, or >5 years), and 3) cumulative dose of medication use. The cumulative duration of medication use was categorized using definitions from previous studies investigating effects of antihypertensive medications (Wiens et al., 2006; Boudreau et al., 2008; Cardwell et al., 2014; Dierssen-Sotos et al., 2017; Cheung et al., 2020).

To account for varying potencies of medications belonging to the same class/subclass when calculating cumulative dose, the prescribed quantity of each medication was represented as a proportion of the WHO’s defined daily dose (DDD) for that medication. All proportions were then summed to provide the total prescribed quantity of the class/subclass (Supplementary Table S1). The DDD is the assumed average maintenance dose per day for a medication used for its main indication in adults (Si et al., 2021). Categorizations of the cumulative dose in this study (0, >0–730, >730–1,825, or >1,825 DDDs) were selected according to the categorizations of the cumulative duration of medication use (Sluggett et al., 2020).

Exposure status for all three metrics was time-dependent; allowing for an individual’s exposure status to vary over the follow-up period. A 1-year lag period was applied to reduce the possibility of reverse causality (Tamim et al., 2007). Any antihypertensive medication that was used in the 1-year lag period prior to end of follow-up was not considered.

### 2.4 Outcomes

The outcomes of the study were incidence of colorectal cancer, overall, as well as incidence of proximal colon cancer, distal colon cancer, and rectal cancer. The study outcomes were not mutually exclusive.

### 2.5 Statistical analysis

In primary analyses, associations of the five classes of antihypertensive medications and their subclasses with overall and site-specific risks of colorectal cancer were examined using time-varying Cox proportional hazard regression models, in which age was the time scale (Canchola et al., 2003). Analyses were adjusted for sex, birth year, baseline neighborhood income quintile, health authority at time of cohort entry, and use of other antihypertensive medication classes (parameterized the same as the primary medication class/subclass of interest). In subclass analyses, users of a given subclass were compared with individuals who were never prescribed that class of antihypertensive medications (Chen et al., 2017). For cumulative duration and cumulative dose, *p*-values for linear trends were calculated by modelling the cumulative exposures as continuous variables.

To determine the impact of further adjustment of potential confounders and predictors that were not captured in PopData, sensitivity analyses were conducted in the subset of individuals who participated in the BCGP. First, covariates adjusted for in the primary analyses of the overall cohort were added to models. Then, additional confounders and predictors captured in the BCGP were added to the models to determine whether the magnitude and direction of estimates changed. Due to the smaller dataset and subsequently, lower number of exposures and outcomes, only associations of ACEIs and ARBs (ever or never) with overall risk of colorectal cancer could be examined.

A second sensitivity analysis was conducted to examine possible confounding by indication. The indications for antihypertensive medications include hypertension, stroke and heart failure (Khalil and Zeltser, 2022). The various indications can lead to individuals who are prescribed a given medication being inherently different from those who are prescribed a different medication. To address this, active comparator analyses were conducted (Lund et al., 2015). Associations between antihypertensive medication classes with overall and site-specific risk of colorectal cancer were calculated for individuals who used one class of antihypertensive medications (*n* = 583,697). This cohort was defined using a time-varying approach where an individuals’ follow-up period started at the time they first used a class of antihypertensive medications. Individuals were censored when they stopped using the class of medications, started using a different class of antihypertensive medications, were diagnosed with cancer, died or moved out of province, whichever occurred first. Each class of antihypertensive medications was compared relative to ARBs.

Participants who had missing information on the sites of their colon cancer were excluded from analyses of proximal and distal colon cancers (*n* = 1,035). Missing values for covariates (up to 13.43%) (Supplementary Table S2) were imputed using single imputation (Zhang, 2016). Hazard ratios (HRs) and associated 95% confidence intervals (95% CIs) were reported for all analyses, which were conducted using R, version R-4.0.5 (Team, 2016).

## 3 Results

A total of 1,693,297 BC residents were included in the study with 21,800,976 person-years of follow up. The mean [standard deviation (SD)] age of participants on their index date (the time of cohort entry) was 53.9 (7.38) years. The proportion of males and females were similar (50.3% vs. 49.7%). Characteristics of the study cohort are presented in Table 1.

**TABLE 1:** Characteristics of the study cohort.

Characteristics	All (*n* = 1,693,297)
Mean (SD)	
Index age	53.9 (7.38)
Frequency (%)	
Index age	
50–59 years	1,398,155 (82.6)
60–69 years	178,812 (10.6)
70–79 years	86,019 (5.08)
80 years or older	30,311 (1.79)
Birth year	
≤1946	656,945 (38.8)
1947–1952	315,292 (18.6)
1953–1958	334,930 (19.8)
1959–1965	386,130 (22.8)
Sex	
Male	851,220 (50.3)
Female	842,077 (49.7)
Income quintile at baseline	
1 (lowest)	321,049 (19.0)
2	323,250 (19.1)
3	332,224 (19.6)
4	347,973 (20.6)
5 (highest)	368,801 (21.8)
Health authority at baseline	
Interior	298,867 (17.7)
Fraser	544,733 (32.2)
Vancouver Coastal	445,337 (26.3)
Vancouver Island	300,729 (17.8)
Northern	103,631 (6.12)

Abbreviations: SD, standard deviation.

Over the follow-up period [mean (SD) = 12.9 (6.37) years], 28,460 individuals were diagnosed with colorectal cancer, among whom 9,366, 7,616, and 10,638 individuals were diagnosed with proximal colon cancer, distal colon cancer, and rectal cancer, respectively.

Associations between antihypertensive medication classes and subclasses as a binary exposure (ever or never) with overall and site-specific risks of colorectal cancer are summarized in Table 2. Ever use of diuretics was significantly associated with 8% higher risk of colorectal cancer (HR 1.08, 95% CI 1.04–1.12). Ever use of diuretics was also associated with increased risks of proximal colon cancer (HR 1.08, 95% CI 1.02–1.14), distal colon cancer (HR 1.08, 95% CI 1.01–1.16), and rectal cancer (HR 1.09, 95% CI 1.02–1.16). In analyses of subclasses of diuretics, the risks of proximal colon cancer and distal colon cancer in individuals who ever used loop diuretics were 1.20 (HR 1.20, 95% CI 1.09–1.32), and 1.13 (HR 1.13, 95% CI 1.01–1.28) relative to nonusers, respectively. No statistically significant associations with the other medication classes were observed.

**TABLE 2:** Associations of antihypertensive medications use (ever/never) with overall and site-specific risks of colorectal cancer^a^.

	All observations	Colorectal cancer		Proximal colon cancer		Distal colon cancer		Rectal cancer	
	*n* = 1,693,297 n (%)	Case n	*n* = 28,460 HR (95% CI)^b^	Case n	*n* = 9,366 HR (95% CI)^b^ ^,^ ^c^	Case n	*n* = 7,616 HR (95% CI)^b^ ^,^ ^c^	Case n	*S* = 10,638 HR (95% CI)^b^
Medication class									
ACEIs									
No	1,257,759 (74.3)	19,552	Reference	6,168	Reference	5,202	Reference	7.637	Reference
Yes	435,538 (25.7)	8.908	1.03 (0.99, 1.07)	3,198	1.05 (0.99, 1.12)	2,414	1.07 (1.00, 1.15)	3,001	1.06 (0.99, 1.13)
ARBs									
No	1,530,294 (90.4)	25,575	Reference	8,228	Reference	6,798	Reference	9,723	Reference
Yes	163,003 (9.63)	2,885	1.03 (0.97, 1.08)	1,078	1.01 (0.93, 1.10)	818	1.08 (0.98, 1.18)	915	0.97 (0.89, 1.06)
BBs									
No	1,386,498 (81.9)	21,950	Reference	6,973	Reference	5,882	Reference	8,462	Reference
Yes	306,799 (18.1)	6,510	0.98 (0.94, 1.02)	2,393	1.00 (0.93, 1.07)	1,734	0.98 (0.91, 1.06)	2,176	0.95 (0.88, 1.02)
CCBs									
No	1,463,608 (86.4)	23,928	Reference	7,646	Reference	6,404	Reference	9,204	Reference
Yes	229,689 (13.6)	4,532	0.99 (0.94, 1.04)	1,720	1.04 (0.97, 1.12)	1,212	0.99 (0.90, 1.08)	1,434	0.94 (0.86, 1.01)
Diuretics									
No	1,194,783 (70.5)	18,125	Reference	5,562	Reference	4.890	Reference	7,221	Reference
Yes	498,514 (29.4)	10,335	1.08 (1.04, 1.12)	3,804	1.08 (1.02, 1.14)	2,726	1.08 (1.01, 1.16)	3,417	1.09 (1.02, 1.16)
Medication subclass^d^									
β1 blockers									
No	1,447,120 (85.5)	21,950	Reference	6,968	Reference	5,876	Reference	8,462	Reference
Yes	246,177 (14.5)	4,805	0.98 (0.94, 1.03)	1,779	1.02 (0.95, 1.10)	1,279	0.96 (0.88, 1.04)	1,604	0.96 (0.89, 1.07)
β1/β2 blockers									
No	1,602,264 (94.6)	21,950	Reference	6,968	Reference	5,876	Reference	8,462	Reference
Yes	91,033 (5.38)	1,110	0.95 (0.89, 1.02)	377	0.90 (0.80, 1.01)	314	0.94 (0.81, 1.08)	383	0.87 (0.77, 1.00)
DHPs									
No	1,505,881 (88.9)	23,928	Reference	7,641	Reference	6,398	Reference	9,204	Reference
Yes	187,416 (11.1)	3,029	0.97 (0.92, 1.03)	1,122	1.01 (0.93, 1.10)	834	0.97 (0.88, 1.20)	965	0.93 (0.85, 1.03)
NDHPs									
No	1,627,837 (96.1)	23,928	Reference	7,641	Reference	6,398	Reference	9,204	Reference
Yes	65,460 (3.87)	1,018	0.91 (0.83, 1.00)	394	1.02 (0.89, 1.16)	261	0.89 (0.76, 1.05)	328	0.83 (0.71, 0.97)
Thiazide diuretics									
No	1,362,856 (80.5)	18,125	Reference	5,557	Reference	4,884	Reference	7,221	Reference
Yes	330,441 (19.5)	4,585	1.04 (1.00, 1.09)	1,591	1.02 (0.95, 1.09)	1,262	1.01 (0.94, 1.09)	1,617	1.07 (1.00, 1.15)
Loop diuretics									
No	1,548,441 (91.4)	18,125	Reference	5.557	Reference	4,884	Reference	7,221	Reference
Yes	144,856 (8.56)	1,519	1.05 (0.98, 1.13)	611	1.20 (1.09, 1.32)	372	1.13 (1.01, 1.28)	459	0.89 (0.78, 1.01)
Potassium sparing diuretics									
No	1,508,420 (89.1)	18,125	Reference	5,557	Reference	4,884	Reference	7,221	Reference
Yes	184,877 (10.9)	1,418	0.94 (0.87, 1.02)	470	0.98 (0.86, 1.11)	414	0.93 (0.80, 1.08)	495	0.93 (0.81, 1.06)

^a^ A 1-year lag period was applied.

^b^ Adjusted for age, sex, birth year, health authority, neighborhood income quintile, and use of other antihypertensive medication classes.

^c^ Observations with missing outcomes were excluded from the analysis (*n* = 1,035).

^d^ In the analyses of antihypertensive subclasses, users of a particular subclass were compared with participants who never used a medication from that class of antihypertensive medications during the study period.

Abbreviations: HR, hazard ratio; 95% CI, 95% confidence interval; ACEI, angiotensin-converting enzyme inhibitor; ARB, angiotensin II, receptor blocker; BB, beta-blocker; CCB, calcium channel blocker; DHP, dihydropyridine; NDHP, non-dihydropyridine.

While there was evidence of a linear trend in the association between cumulative duration of ARB use and rectal cancer risk (*p*-value = 0.04), the categorical analyses did not support such a relationship (Table 3). No other significant associations with cumulative duration or cumulative dose (Table 4) were observed.

**TABLE 3:** Associations between the cumulative duration of antihypertensive medication use with overall and site-specific risks of colorectal cancer^a^ ^,^ ^b^.

	Colorectal cancer		Proximal colon cancer		Distal colon cancer		Rectal cancer	
	Case n	*n* = 28,460 HR (95% CI)^c^	Case n	*n* = 9,366 HR (95% CI)^c^ ^,^ ^d^	Case n	*n* = 7,616 HR (95% CI)^c^ ^,^ ^d^	Case n	*n* = 10,638 HR (95% CI)^c^
Medication class								
ACEIs								
0	19,552	Reference	6,168	Reference	5,202	Reference	7,637	Reference
>0–2	4,149	1.03 (0.99, 1.07)	1,428	1.07 (1.00, 1.14)	1,147	0.99 (0.91, 1.07)	1,398	1.01 (0.94, 1.08)
>2–5	2,101	0.97 (0.93, 1.01)	704	1.02 (0.96, 1.09)	581	0.93 (0.87, 1.01)	685	0.93 (0.87, 1.00)
>5	2,658	1.02 (0.97, 1.04)	1,012	1.06 (0.98, 1.14)	685	1.03 (0.95, 1.11)	918	0.97 (0.91, 1.05)
P trend^e^		0.30		0.08		0.77		0.68
ARBs								
0	25,575	Reference	8,228	Reference	6,798	Reference	9,723	Reference
>0–2	1,043	0.98 (0.93, 1.04)	404	1.04 (0.95, 1.14)	288	0.93 (0.83, 1.04)	364	0.93 (0.84, 1.04)
>2–5	786	0.97 (0.91, 1.03)	305	1.01 (0.92, 1.12)	249	0.91 (0.81, 1.03)	240	0.99 (0.88, 1.11)
>5	1,056	0.96 (0.89, 1.03)	429	0.92 (0.82, 1.04)	281	0.96 (0.84, 1.10)	72,269	1.00 (0.88, 1.14)
P trend^e^		0.38		0.81		0.64		0.04
BBs								
0	21,950	Reference	6,973	Reference	5,882	Reference	8,462	Reference
>0–2	2,301	0.97 (0.92, 1.01)	1,130	0.96 (0.89, 1.04)	889	0.95 (0.87, 1.04)	1,039	0.98 (0.90, 1.06)
>2–5	767	1.02 (0.97, 1.07)	591	1.04 (0.96, 1.12)	439	0.99 (0.91, 1.09)	507	1.04 (0.96, 1.13)
>5	884	0.97 (0.92, 1.02)	672	0.99 (0.91, 1.07)	446	0.97 (0.88, 1.06)	540	0.96 (0.88, 1.06)
P trend^e^		0.05		0.24		0.32		0.17
CCBs								
0	23,928	Reference	7,646	Reference	6,404	Reference	9,204	Reference
>0–2	2,068	1.01 (0.96, 1.07)	774	1.08 (0.99, 1.17)	571	0.94 (0.85, 1.04)	647	0.99 (0.90, 1.09)
>2–5	1,177	1.02 (0.96, 1.08)	426	1.06 (0.97, 1.16)	334	0.89 (0.80, 0.99)	375	1.06 (0.96, 1.17)
>5	1,287	0.97 (0.91, 1.03)	520	1.03 (0.94, 1.13)	307	0.94 (0.85, 1.05)	411	0.92 (0.82, 1.02)
P trend^e^		0.98		0.13		0.26		0.46
Diuretics								
0	18,125	Reference	5,562	Reference	4,890	Reference	7,221	Reference
>0–2	5,469	1.02 (0.97, 1.06)	1,965	0.99 (0.93, 1.06)	1,453	1.05 (0.97, 1.14)	1,845	1.03 (0.95, 1.11)
>2–5	2,293	1.00 (0.96, 1.04)	852	0.97 (0.91, 1.03)	598	1.02 (0.95, 1.10)	742	1.01 (0.95, 1.09)
>5	2,573	0.98 (0.94, 1.02)	987	0.97 (0.91, 1.03)	675	1.01 (0.93, 1.09)	827	0.97 (0.91, 1.05)
P trend^e^		0.14		0.31		0.44		0.22
β1 blockers								
0	21,950	Reference	6,968	Reference	5,876	Reference	8,462	Reference
>0–2	2,189	0.97 (0.92, 1.02)	801	0.97 (0.90, 1.05)	581	0.94 (0.85, 1.04)	738	0.99 (0.90, 1.08)
>2–5	1,255	1.05 (0.99, 1.11)	458	1.03 (0.95, 1.12)	339	1.04 (0.94, 1.15)	417	1.08 (0.98, 1.18)
>5	1,361	0.96 (0.90, 1.02)	520	0.97 (0.89, 1.06)	359	0.98 (0.88, 1.09)	449	0.95 (0.86, 1.05)
P trend^e^		0.06		0.44		0.26		0.13
β1/β2 blockers								
0	21,950	Reference	6,968	Reference	5,876	Reference	8,462	Reference
>0–2	748	0.89 (0.78, 1.00)	258	0.86 (0.71, 1.04)	208	0.90 (0.71, 1.13)	260	0.90 (0.73, 1.11)
>2–5	214	1.04 (0.93, 1.17)	68	1.06 (0.88, 1.27)	66	0.96 (0.78, 1.18)	70	1.15 (0.94, 1.41)
>5	148	0.96 (0.86, 1.06)	51	0.96 (0.81, 1.14)	40	0.90 (0.75, 1.09)	53	1.04 (0.86, 1.26)
P trend^e^		0.06		0.12		0.36		0.08
DHPs								
0	23,928	Reference	7,641	Reference	6,398	Reference	9,204	Reference
>0–2	1,366	1.02 (0.96, 1.09)	497	1.06 (0.96, 1.16)	391	0.93 (0.82, 1.04)	423	1.04 (0.94, 1.16)
>2–5	816	1.02 (0.95, 1.09)	297	1.04 (0.94, 1.15)	233	0.89 (0.79, 1.00)	260	1.09 (0.97, 1.22)
>5	847	0.95 (0.89, 1.02)	328	1.00 (0.90, 1.11)	210	0.96 (0.85, 1.08)	282	0.88 (0.78,1.00)
P trend^e^		0.88		0.32		0.17		0.88
NDHPs								
0	23,928	Reference	7,641	Reference	6,398	Reference	9,204	Reference
>0–2	538	0.92 (0.83, 1.01)	210	1.03 (0.89, 1.19)	141	0.89 (0.74, 1.07)	171	0.85 (0.71, 1.01)
>2–5	223	1.11 (1.00, 1.24)	72	1.14 (0.98, 1.34)	62	1.02 (0.84, 1.24)	78	1.22 (1.00, 1.50)
>5	258	1.10 (0.98, 1.24)	112	1.13 (0.96, 1.35)	58	0.96 (0.78, 1.17)	79	1.21 (0.98, 1.51)
P trend^e^		0.22		0.83		0.27		0.11
Thiazide diuretics								
0	18,125	Reference	5,557	Reference	4,884	Reference	7,221	Reference
>0–2	2,113	1.01 (0.96, 1.06)	738	0.99 (0.92, 1.07)	583	1.03 (0.94, 1.12)	743	1.02 (0.94, 1.10)
>2–5	1,163	1.01 (0.96, 1.05)	395	0.97 (0.90, 1.04)	313	1.05 (0.97, 1.14)	420	1.01 (0.93, 1.09)
>5	1,309	0.99 (0.94, 1.04)	458	0.98 (0.92, 1.06)	393	1.04 (0.95, 1.13)	454	0.97 (0.89, 1.05)
P trend^e^		0.65		0.98		0.96		0.53
Loop diuretics								
0	18,125	Reference	5,557	Reference	4,884	Reference	7,221	Reference
>0–2	933	1.02 (0.91, 1.13)	365	1.08 (0.93, 1.25)	238	1.05 (0.86, 1.27)	286	0.94 (0.77, 1.15)
>2–5	316	1.07 (0.97, 1.18)	133	1.06 (0.92, 1.21))	74	1.01 (0.85, 1.21)	93	1.08 (0.90, 1.30)
>5	370	1.08 (0.99, 1.19)	113	1.14 (1.00, 1.31)	60	1.09 (0.92, 1.29)	80	1.05 (0.88, 1.24)
P trend^e^		0.57		0.74		0.67		0.82
Potassium sparing diuretics								
0	18,125	Reference	5,557	Reference	4,884	Reference	7,221	Reference
>0–2	1,162	1.16 (1.00, 1.34)	390	1.22 (0.98, 1.52)	322	1.16 (0.87, 1.54)	425	1.13 (0.87, 1.47)
>2–5	127	1.16 (0.99, 1.36)	56	1.25 (1.00, 1.45)	31	1.15 (0.89, 1.48)	31	1.30 (0.99, 1.70)
>5	139	1.01 (0.88, 1.16)	49	1.05 (0.86, 1.29)	61	0.88 (0.70, 1.11)	39	1.03 (0.81, 1.31)
P trend^e^		0.54		0.15		0.77		1.00

^a^ A 1-year lag period was applied.

^b^ The duration unit is 1 years.

^c^ Adjusted for age, sex, birth year, health authority, neighborhood income quintile, and use durations of other antihypertensive medication classes.

^d^ Observations with missing outcomes were excluded from the analysis (*n* = 1,035).

^e^ *p*-value for trend was the *p*-value for the cumulative exposure variable modelled as continuous.

^f^ In the analyses of antihypertensive subclasses, users of a particular subclass were compared with participants who were never prescribed that class of antihypertensive medications.

Abbreviations: HR, hazard ratio; 95% CI, 95% confidence interval; ACEI, angiotensin-converting enzyme inhibitor; ARB, angiotensin II, receptor blocker; BB, beta-blocker; CCB, calcium channel blocker; DHP, dihydropyridine; NDHP, non-dihydropyridine.

**TABLE 4:** Associations between the cumulative dose of antihypertensive medications with overall and site-specific risks of colorectal cancer^a^ ^,^ ^b^.

	Colorectal cancer		Proximal colon cancer		Distal colon cancer		Rectal cancer	
	Case n	*n* = 28,460	Case n	*n* = 9,366	Case n	*n* = 7,616	Case n	*n* = 10,638
		HR (95% CI)^c^		HR (95% CI)^c^ ^,^ ^d^		HR (95% CI)^c^ ^,^ ^d^		HR (95% CI)^c^
Medication class								
ACEIs								
0	19,552	Reference	6,168	Reference	5,202	Reference	7,637	Reference
>0–730	3,560	1.03 (0.99, 1.07)	1,281	1.06 (1.00, 1.12)	978	1.06 (0.99, 1.13)	1,183	1.06 (1.00, 1.13)
>730–1,825	1,724	0.99 (0.95, 1.03)	588	1.02 (0.95, 1.10)	480	0.98 (0.90, 1.06)	608	0.96 (0.90, 1.04)
>1,825	3,624	0.99 (0.94, 1.04)	1,329	1.03 (0.95, 1.11)	956	0.99 (0.90, 1.08)	1,219	0.95 (0.87, 1.03)
P trend^e^		0.46		0.06		0.88		0.30
ARBs								
0	25,575	Reference	8,228	Reference	6,798	Reference	9,723	Reference
>0–730	1,097	1.01 (0.95, 1.07)	405	1.07 (0.97, 1.17)	315	0.95 (0.85, 1.06)	354	0.96 (0.87, 1.07)
>730–1,825	686	1.01 (0.94, 1.08)	279	1.04 (0.93, 1.16)	185	1.03 (0.90, 1.17)	233	0.98 (0.87, 1.11)
>1,825	1,102	1.03 (0.95, 1.12)	454	1.01 (0.89, 1.14)	309	1.13 (0.97, 1.32)	333	0.97 (0.84, 1.11)
P trend^e^		0.44		0.41		0.51		0.34
BBs								
0	21,950	Reference	6,973	Reference	5,882	Reference	8,462	Reference
>0–730	3,818	1.02 (0.95, 1.09)	1,361	1.04 (0.93, 1.15)	1,017	0.97 (0.86, 1.11)	1,293	1.05 (0.93, 1.18)
>730–1,825	1,516	1.06 (1.00, 1.13)	536	1.07 (0.97, 1.18)	404	1.02 (0.91, 1.15)	454	1.13 (1.01, 1.25)
>1,825	1,176	0.99 (0.93, 1.05)	406	0.96 (0.88, 1.06)	141	0.96 (0.87, 1.07)	357	1.02 (0.92, 1.13)
P trend^e^		0.87		0.74		0.93		0.62
CCBs								
0	23,928	Reference	7,646	Reference	6,404	Reference	9,204	Reference
>0–730	2,088	1.03 (0.97, 1.08)	788	1.07 (0.98, 1.16)	579	0.96 (0.87, 1.06)	643	1.03 (0.94, 1.13)
>730–1,825	1,091	1.04 (0.98, 1.10)	400	1.05 (0.96, 1.14)	302	0.92 (0.83, 1.02)	345	1.10 (0.99, 1.22)
>1,825	1,353	0.97 (0.91, 1.03)	532	1.00 (0.90, 1.10)	331	0.95 (0.85, 1.06)	446	0.94 (0.84, 1.05)
P trend^e^		0.23		0.07		0.38		0.33
Diuretics								
0	18,125	Reference	5,562	Reference	4,890	Reference	7,221	Reference
>0–730	5,895	1.00 (0.96, 1.06)	2,152	1.01 (0.93, 1.09)	1,553	1.02 (0.93, 1.11)	1,970	0.99 (0.91, 1.08)
>730–1,825	2,329	0.96 (0.92, 1.01)	850	0.98 (0.91, 1.04)	623	0.95 (0.87, 1.03)	766	1.01 (0.93, 1.09)
>1,825	2,111	0.97 (0.93, 1.02)	802	0.98 (0.92, 1.05)	550	0.95 (0.88, 1.03)	681	0.98 (0.90, 1.05)
P trend^e^		0.23		0.12		0.42		0.14
Medication subclass^f^								
β1 blockers								
0	21,950	Reference	6,968	Reference	5,876	Reference	8,462	Reference
>0–730	2,889	1.01 (0.94, 1.09)	337	1.04 (0.93, 1.16)	767	0.96 (0.83, 1.10)	970	1.04 (0.92, 1.17)
>730–1,825	1,097	1.09 (1.02, 1.17)	417	1.07 (0.97, 1.19)	292	1.04 (0.92, 1.18)	380	1.09 (0.97, 1.23)
>1,825	819	0.98 (0.92, 1.04)	305	0.95 (0.87, 1.05)	220	0.95 (0.85, 1.06)	275	1.02 (0.91, 1.13)
P trend^e^		0.79		0.66		0.98		0.37
β1/β2 blockers								
0	21,950	Reference	6,968	Reference	5,876	Reference	8,462	Reference
>0–730	620	0.98 (0.78, 1.24)	202	0.99 (0.70, 1.41)	170	0.96 (0.63, 1.48)	208	0.97 (0.64, 1.45)
>730–1,825	288	0.97 (0.79, 1.19)	88	1.06 (0.77, 1.46)	74	0.90 (0.62, 1.31)	95	0.91 (0.64, 1.30)
>1,825	265	0.84 (0.71, 1.00)	87	0.92 (0.69, 1.23)	70	0.79 (0.58, 1.07)	80	0.76 (0.57, 1.02)
P trend^e^		0.25		0.31		0.43		0.76
DHPs								
0	23,928	Reference	7,641	Reference	6,398	Reference	9,204	Reference
>0–730	1,339	1.04 (0.98, 1.10)	493	1.07 (0.97, 1.17)	383	0.96 (0.86, 1.08)	411	1.07 (0.97, 1.19)
>730–1,825	733	1.07 (1.00, 1.14)	261	1.07 (0.97, 1.18)	215	0.93 (0.83, 1.05)	230	1.17 (1.04, 1.31)
>1,825	955	0.97 (0.91, 1.05)	368	0.99 (0.88, 1.10)	236	0.99 (0.87, 1.12)	324	0.92 (0.81, 1.05)
P trend^e^		0.22		0.19		0.32		0.13
NDHPs								
0	23,928	Reference	7,641	Reference	6,398	Reference	9,204	Reference
>0–730	577	0.91 (0.82, 1.02)	225	1.01 (0.86, 1.18)	154	0.88 (0.72, 1.09)	178	0.84 (0.69, 1.02)
>730–1,825	227	1.10 (0.98, 1.23)	72	1.08 (0.92, 1.28)	56	1.02 (0.83, 1.25)	80	1.16 (0.94, 1.42)
Thiazide diuretics								
0	18,125	Reference	55,571	Reference	4,884	Reference	7,221	Reference
>0–730	2,462	0.99 (0.94, 1.05)	853	0.99 (0.91, 1.08)	671	1.00 (0.91, 1.11)	875	0.97 (0.88, 1.07)
>730–1,825	1,148	0.99 (0.94, 1.04)	400	0.98 (0.91, 1.06)	320	0.99 (0.91, 1.09)	401	0.99 (0.91, 1.08)
>1,825	975	0.97 (0.92, 1.02)	338	0.96 (0.89, 1.04)	271	0.99 (0.90, 1.08)	341	0.98 (0.90, 1.07)
P trend^e^		0.66		0.94		0.91		0.69
Loop diuretics								
0	18,125	Reference	5,557	Reference	4,884	Reference	7,221	Reference
>0–730	954	1.05 (0.94, 1.17)	378	1.16 (0.99, 1.35)	237	1.01 (0.82, 1.24)	288	1.03 (0.85, 1.25)
>730–1,825	299	1.08 (0.97, 1.19)	118	1.10 (0.95, 1.27)	72	0.94 (0.78, 1.06)	89	1.19 (0.99, 1.43)
>1,825	266	1.08 (0.98, 1.19)	114	1.14 (0.99, 1.31)	63	1.01 (0.85, 1.20)	82	1.09 (0.91, 1.30)
P trend^e^		0.09		0.13		0.44		0.37
Potassium sparing diuretics								
0	18,125	Reference	5,557	Reference	5,884	Reference	7,221	Reference
>0–730	952	1.30 (0.90, 1.88)	342	1.45 (0.84, 2.50)	271	1.19 (0.55, 2.26)	328	1.18 (0.60, 2.30)
>730–1,825	234	1.20 (0.88, 1.63)	75	1.19 (0.76, 1.88)	72	0.96 (0.52, 1.79)	86	1.26 (0.72, 2.21)
>1,825	221	0.89 (0.70, 1.11)	73	0.92 (0.66, 1.29)	71	0.73 (0.49, 1.11)	81	0.88 (0.58, 1.34)
P trend^e^		0.23		0.21		0.34		0.74

^a^ A 1-year lag period was applied.

^b^ The dose unit is one defined daily dose.

^c^ Adjusted for age, sex, birth year, health authority, neighborhood income quintile, and cumulative dose of other antihypertensive medication classes.

^d^ Observations with missing outcomes were excluded from the analysis (*n* = 1,035).

^e^ *p*-value for trend was the *p*-value for the cumulative exposure variable modelled as continuous.

^f^ In the analyses of antihypertensive subclasses, users of a particular subclass were compared with participants who were never prescribed that class of antihypertensive medications.

Abbreviations: HR, hazard ratio; 95% CI, 95% confidence interval; ACEI, angiotensin-converting enzyme inhibitor; ARB, angiotensin II, receptor blocker; BB, beta-blocker; CCB, calcium channel blocker; DHP, dihydropyridine; NDHP, non-dihydropyridine.

Results of sensitivity analyses using data from the BCGP cohort (*n* = 19,819) are presented in Table 5. Model 1 includes covariates adjusted for in the primary analyses of administrative health data, while Model 2 includes additional lifestyle and health related variables not captured in the administrative health data. Similar to the primary analyses, ever use of ACEIs and ARBs were not associated with overall risk of colorectal cancer (Model 1, HR 1.18, 95% CI 0.71–1.95 and HR 1.18, 95% CI 0.73–1.89, respectively). Although the magnitudes of the estimates were larger than the corresponding primary analyses, the direction (positive) and lack of statistical significance of estimates were consistent. The results remained similar with further adjustment for lifestyle and health variables in Model 2 (HR 1.23, 95% CI 0.34–3.67 and HR 1.57, 95% CI 0.51–4.33, respectively). The active comparator analysis did not find any differences in overall or site-specific risk of colorectal cancer comparing users of other classes and subclasses of antihypertensive medications with individuals who used ARBs (Table 6).

**TABLE 5:** Association of use of angiotensin-converting enzyme inhibitors and angiotensin II receptor blockers (ever or never) with overall risk of colorectal cancer in a subset of participants who were part of the British Columbia Generations Project^a^.

Medication	All observations	Colorectal cancer (*n* = 233)		
	(*n* = 19,819)	Case	Model 1	Model 2
	N (%)	n	HR (95% CI)^b^	HR (95% CI)^c^
ACEIs				
No	16,776 (84.65)	186	Reference	Reference
Yes	3,043 (15.35)	47	1.18 (0.71, 1.95)	1.23 (0.34, 3.67)
ARBs				
No	18,507 (93.38)	213	Reference	Reference
Yes	1,312 (6.62)	20	1.18 (0.73, 1.89)	1.57 (0.51, 4.33)

^a^ A 1-year lag period was applied.

^b^ Adjusted for age, sex, birth year, health authority, neighborhood income quintile, and use of other antihypertensive medication classes.

^c^ Adjusted for age, sex, birth year, health authority, ethnicity, education, marital status, household income, body mass index, family cancer history, vegetable ypertensive medication classes.

Abbreviations: HR, hazard ratio; 95% CI, 95% confidencand fruit consumption, alcohol consumption, smoking status, moderate-to-vigorous physical activity, fecal occult blood test, sigmoidoscopy or colonoscopy, polyp removed, and use of other antihe interval; ACEI, angiotensin-converting enzyme inhibitor; ARB, angiotensin II, receptor blocker.

**TABLE 6:** Overall and site-specific risks of colorectal cancer among monotherapy users of antihypertensive medications^a^.

Medication use	All observations	Colorectal cancer		Proximal colon cancer		Distal colon cancer		Rectal cancer	
	*n* = 583,697	Case	*n* = 1,171	Case	*n* = 394	Case	*n* = 307	Case	*n* = 467
	N (%)	n	HR (95% CI)^b^	n	HR (95% CI)^b^ ^,^ ^c^	n	HR (95% CI)^b^ ^,^ ^c^	n	HR (95% CI)^b^
Medication class									
ARBs	25,800 (4.42)	146	Reference	42	Reference	47	Reference	57	Reference
ACEIs	151,077 (25.88)	369	1.05 (0.91, 1.22)	128	1.14 (0.88, 1.47)	93	0.89 (0.69, 1.14)	148	1.14 (0.89, 1.47)
BBs	113,676 (19.48)	183	0.92 (0.79, 1.06)	63	0.95 (0.74, 1.23)	50	0.82 (0.64, 1.06)	69	0.97 (0.75, 1.25)
CCBs	46,705 (8.00)	72	0.98 (0.83, 1.15)	25	1.10 (0.83, 1.44)	18	0.88 (0.67, 1.16)	29	0.98 (0.74, 1.30)
Diuretics	246,439 (42.22)	379	0.94 (0.81, 1.08)	136	1.00 (0.78, 1.28)	99	0.82 (0.64, 1.05)	144	1.01 (0.79, 1.30)
Medication subclass^d^									
β1 blockers	63,717 (10.92)	98	0.94 (0.80, 1.10)	36	1.01 (0.78, 1.32)	29	0.86 (0.66, 1.12)	33	0.94 (0.72, 1.22)
β1/β2 blockers	46,735 (8.01)	56	0.88 (0.75, 1.04)	21	0.85 (0.65, 1.12)	15	0.79 (0.60, 1.04)	20	1.01 (0.77, 1.32)
DHPs	31,320 (5.37)	39	0.97 (0.81, 1.15)	14	1.06 (0.79, 1.43)	12	0.91 (0.68, 1.23)	13	0.96 (0.71, 1.30)
NDHPs	14,693 (2.52)	22	1.00 (0.82, 1.20)	8	1.13 (0.83, 1.55)	6	0.86 (0.62, 1.19)	8	1.01 (0.73, 1.40)
Thiazide diuretics	93,345 (15.99)	181	1.02 (0.88, 1.19)	67	1.06 (0.82, 1.37)	49	0.87 (0.68, 1.13)	65	1.14 (0.89, 1.47)
Loop diuretics	30,278 (5.19)	57	0.96 (0.81, 1.13)	25	1.13 (0.86, 1.48)	15	0.84 (0.63, 1.11)	18	0.97 (0.73, 1.28)
Potassium sparing diuretics	94,841 (16.25)	136	0.88 (0.76, 1.01)	47	0.94 (0.73, 1.22)	38	0.80 (0.63, 1.02)	51	0.88 (0.68, 1.14)

^a^ A 1-year lag period was applied.

^b^ Adjusted for age, sex, birth year, health authority, and neighborhood income quintile.

^c^ Observations with missing outcomes were excluded from the analysis (*n* = 6).

^d^ Each subclass analysis was performed among a subcohort including monotherapy users of a subclass of antihypertensive medications and monotherapy users of other four classes of antihypertensive medications.

Abbreviations: HR, hazard ratio; 95% CI, 95% confidence interval; ARB, angiotensin II, receptor blocker; ACEI, angiotensin-converting enzyme inhibitor; BB, beta-blocker; CCB, calcium channel blocker; DHP, dihydropyridine; NDHP, non-dihydropyridine.

## 4 Discussion

In this large population-based study, associations between five major classes of antihypertensive medications and their subclasses were comprehensively examined with overall and site-specific risks of colorectal cancer. We did not find any compelling evidence of associations of ACEIs, ARBs, BBs, and CCBs, or their subclasses with overall and site-specific risks of colorectal cancer. Findings suggested associations between ever use of diuretics and risks of overall and site-specific colorectal cancers and associations between ever use of loop diuretics and risks of proximal and distal colon cancers, but not rectal cancer. However, associations were not observed when exposure was measured as cumulative duration and dose, which captures exposure more precisely.

Multiple studies have examined associations between diuretic medication use and colorectal cancer risk, with most indicating a null association (Boudreau et al., 2008; Makar et al., 2014; Mackenzie et al., 2017; Cheung et al., 2020; Cho et al., 2021). Only two studies have examined the association of loop diuretics with colon cancer, and their findings were inconsistent (Tenenbaum et al., 2001; Deshpande, 2013). A case-control study conducted by Deshpande (2013) suggested that patients with colon cancer were more likely to have used loop diuretic medication than people without colon cancer (OR 1.29, 95% CI 1.21–1.37). Results from a cohort study, on the other hand, indicated that loop diuretics were not associated with risk of colon cancer (HR 1.5, 95% CI 0.8–2.7), although participants of the study were individuals with a previous myocardial infarction or stable angina, which limited the generalizability of the study (Tenenbaum et al., 2001).

Ten studies have examined associations of ACEIs and ARBs with colorectal risk (Boudreau et al., 2008; van der Knaap et al., 2008; Chang et al., 2011; Hallas et al., 2012; Deshpande, 2013; Wang et al., 2013; Makar et al., 2014; Dierssen-Sotos et al., 2017; Cheung et al., 2020; Cho et al., 2021). The null association of ACEIs and ARBs with colorectal cancer risk in this study aligns with eight of the prior studies (Boudreau et al., 2008; van der Knaap et al., 2008; Chang et al., 2011; Hallas et al., 2012; Makar et al., 2014; Dierssen-Sotos et al., 2017; Cho et al., 2021). None of the previous studies assessed the dose of ACEI or ARB use, and thus our findings provide further evidence of a null association. In contrast, two case-control studies suggested increased risk of colorectal cancer. The case-control study conducted by Deshpande (2013) reported that ACEIs and ARBs were associated with modest increased risk of colorectal cancer (OR 1.07, 95% CI 1.02–1.12 and OR 1.07, 95% CI 1.02–1.12, respectively). Hallas et al. (2012) found a positive association between use of ACEIs and colorectal cancer risk (OR 1.30, 95% CI 1.22–1.39). Neither of the case-control studies considered changes in medication use or doses over time. The retrospective cohort study conducted by Wang et al. suggests inverse associations between ARB use and colorectal cancer risk (HR 0.68, 95% CI 0.56–0.83) (Wang et al., 2013). The study had a short follow-up time (mean = 4.8 years) (Wang et al., 2013), which is shorter than the general latency period (5–10 years) for development of colorectal cancer (Haggar and Boushey, 2009).

With the exception of one study (Chang et al., 2015), epidemiologic studies do not support any association between use of BBs and colorectal cancer risk (Jansen et al., 2012; Deshpande, 2013; Makar et al., 2014; Cho et al., 2021), which aligns with the findings of our study. The retrospective cohort study conducted by Chang et al. reported that BB use was associated with decreased risk of colorectal cancer (HR 0.68; 95% CI 0.49–0.93) (Chang et al., 2015). Chang et al. did not exclude prevalent BB users to minimize the influence of previous BB use. Moreover, the mean follow-up years were 6.96 and 6.50 for the exposed and reference cohorts, respectively (Chang et al., 2015). The short follow-up periods may not capture the latency period of colorectal cancer (Haggar and Boushey, 2009). To our knowledge, our study is the only one to assess the dose of BB use, and thus our findings increase confidence in the null association between BB use and colorectal cancer risk.

Most existing studies suggest that there is no association between use of CCBs and colorectal cancer risk (Boudreau et al., 2008; Makar et al., 2014; Cheung et al., 2020; Cho et al., 2021). The one exception is the case-control study by Deshpande (2013), which reported higher colorectal cancer risk among CCB users (OR 1.17, 95% CI 1.11–1.24) relative to non-users. However, Deshpande et al. did not assess the dose and duration of CCB use or changes in CCB use over time. The findings of this study support the overall body of evidence from epidemiologic studies and provide further evidence that the association is null even with prolonged duration and across a range of CCB dose.

The study has several strengths. It is the first to explore associations between antihypertensive medications and colorectal cancer risk using time-varying analytical methods. Using time-dependent exposure measurements that allow individuals’ exposure status to vary over time can provide more statistical power for effect detection, and minimize the likelihood of biases, such as exposure misclassification (Flegal et al., 1986). Time-varying methods also better reflect real-world scenarios where medication use is usually dynamic (Stricker and Stijnen, 2010). The new-user study design is also a strength which minimizes the influence of previous use of antihypertensive medications and increases the accuracy of measures of medication use. The long follow-up period (mean = 12.9 years) is more likely to capture the latency period of colorectal cancer (Haggar and Boushey, 2009). The detailed medication data allowed us to assess duration and dose medication use, which is an unique contribution to the field (Qi et al., 2022). Given the large size of the study (*n* = 1,693,297), we were able to explore associations between antihypertensive medications and site-specific risks of colorectal cancer, which have been rarely investigated in previous studies (Qi et al., 2022). Limited covariates were adjusted for in the primary analyses due to the nature of administrative health data that generally does not comprehensively capture lifestyle, demographic information, and cancer risk factors. Although sensitivity analyses in the BCGP suggested consistency of findings with additional adjustment for lifestyle and health variables, due to the small sample size of the BCGP cohort, associations of BBs, CCBs, and diuretics with colorectal cancer risk could not be examined. As a result, it is possible effect estimates were influenced by residual confounding, particularly for estimates of medication use that are used for specific indications (e.g., loop diuretics and chronic kidney disease). However, the confounding by indication analyses found no differences in colorectal cancer risk for classes and subclasses of antihypertensive medication relative to users of ARBs. As is the case for all prescription database studies, measurements of antihypertensive medications were based on dispensed prescriptions rather than antihypertensive medications actually taken by individuals. While prescriptions and adherence are correlated (Vik et al., 2004), it is possible this led to exposure misclassification which would bias the findings toward to the null.

In conclusion, the study suggests that the most commonly prescribed anti-hypertensive medication classes (ACEIs, ARBs, BBs, CCBs, and diuretics) and subclasses are not associated with risk of colorectal cancer. The study provides evidence for safety of the commonly prescribed antihypertensive medications with respect to colorectal cancer risk.

## Data Availability

The datasets presented in this article are not readily available. Due to legal, ethical or privacy restrictions of Population Data BC (PopData), data used in the study cannot be shared. Privacy policies of PopData are shown in https://www.popdata.bc.ca/privacy/policies. Data access is only available upon request with permission of PopData. Requests to access the datasets should be directed to dataaccess@popdata.bc.ca.

## References

[B1] Ark T. K. Kesselring S. Hills B. McGrail K. (2019). Population Data BC: supporting population data science in British Columbia. Int. J. Popul. data Sci. 4 (2), 1133. 10.23889/ijpds.v5i1.1133 PMC7480325 32935036

[B2] Assimes T. L. Elstein E. Langleben A. Suissa S. (2008). Long‐term use of antihypertensive drugs and risk of cancer. Pharmacoepidemiol. drug Saf. 17 (11), 1039–1049. 10.1002/pds.1656 18780400

[B3] Boudreau D. M. Koehler E. Rulyak S. J. Haneuse S. Harrison R. Mandelson M. T. (2008). Cardiovascular medication use and risk for colorectal cancer. Cancer Epidemiol. Biomarkers Prev. 17 (11), 3076–3080. 10.1158/1055-9965.EPI-08-0095 18957524 PMC2675612

[B4] Brenner D. R. Weir H. K. Demers A. A. Ellison L. F. Louzado C. Shaw A. (2020). Projected estimates of cancer in Canada in 2020. Can. Med. Assoc. J. 192 (9), E199–E205. 10.1503/cmaj.191292 32122974 PMC7055947

[B5] Canchola A. J. Stewart S. L. Bernstein L. West D. W. Ross R. K. Deapen D. (2003). Cox regression using different time-scales. San Francisco, California: Western Users of SAS Software.

[B6] Cardwell C. R. Mc Menamin Ú. C. Hicks B. M. Hughes C. Cantwell M. M. Murray L. J. (2014). Drugs affecting the renin-angiotensin system and survival from cancer: a population based study of breast, colorectal and prostate cancer patient cohorts. BMC Med. 12 (1), 28–15. 10.1186/1741-7015-12-28 24521426 PMC3926686

[B7] Chang C.-H. Lin J.-W. Wu L.-C. Lai M.-S. (2011). Angiotensin receptor blockade and risk of cancer in type 2 diabetes mellitus: a nationwide case-control study. J. Clin. Oncol. 29 (22), 3001–3007. 10.1200/JCO.2011.35.1908 21690476

[B8] Chang P.-Y. Huang W.-Y. Lin C.-L. Huang T.-C. Wu Y.-Y. Chen J.-H. (2015). Propranolol reduces cancer risk: a population-based cohort study. Medicine 94 (27), e1097. 10.1097/MD.0000000000001097 26166098 PMC4504645

[B9] Chen L. Chubak J. Boudreau D. M. Barlow W. E. Weiss N. S. Li C. I. (2017). Use of antihypertensive medications and risk of adverse breast cancer outcomes in a SEER–medicare population. Cancer Epidemiol. Biomarkers Prev. 26 (11), 1603–1610. 10.1158/1055-9965.EPI-17-0346 28807926 PMC5668179

[B10] Cheung K. S. Chan E. W. Seto W. K. Wong I. C. Leung W. K. (2020). ACE (angiotensin-converting enzyme) inhibitors/angiotensin receptor blockers are associated with lower colorectal cancer risk: a territory-wide study with propensity score analysis. Hypertension 76 (3), 968–975. 10.1161/HYPERTENSIONAHA.120.15317 32623923

[B11] Cho I.-J. Shin J.-H. Jung M.-H. Kang C. Y. Hwang J. Kwon C. H. (2021). Antihypertensive drugs and the risk of cancer: a nationwide cohort study. J. Clin. Med. 10 (4), 771. 10.3390/jcm10040771 33671916 PMC7918966

[B12] Clinton S. K. Giovannucci E. L. Hursting S. D. (2020). The world cancer research fund/American institute for cancer research third expert report on diet, nutrition, physical activity, and cancer: impact and future directions. J. Nutr. 150 (4), 663–671. 10.1093/jn/nxz268 31758189 PMC7317613

[B13] De Oliveira A. T. T. Matos D. Logullo A. F. da Silva S. R. M. Neto R. A. Longatto Filho A. (2009). MET Is highly expressed in advanced stages of colorectal cancer and indicates worse prognosis and mortality. Anticancer Res. 29 (11), 4807–4811. 20032439

[B14] Deshpande G. (2013). Association between cardiovascular drugs and colon cancer. Baltimore: University of Maryland.

[B15] Dhalla A. McDonald T. E. Gallagher R. P. Spinelli J. J. Brooks-Wilson A. R. Lee T. K. (2019). Cohort profile: the British Columbia Generations project (BCGP). Int. J. Epidemiol. 48 (2), 377–378k. 10.1093/ije/dyy160 30169793

[B16] Dierssen-Sotos T. Gómez-Acebo I. Palazuelos C. Rodriguez-Moranta F. Pérez-Gómez B. Vazquez J. P. F. (2017). Relationship between drugs affecting the renin-angiotensin system and colorectal cancer: the MCC-Spain study. Prev. Med. 99, 178–184. 10.1016/j.ypmed.2017.01.011 28131779

[B17] Flegal K. M. Brownie C. Haas J. (1986). The effects of exposure misclassification on estimates of relative risk. Am. J. Epidemiol. 123 (4), 736–751. 10.1093/oxfordjournals.aje.a114294 3953551

[B18] Haggar F. A. Boushey R. P. (2009). Colorectal cancer epidemiology: incidence, mortality, survival, and risk factors. Clin. colon rectal Surg. 22 (04), 191–197. 10.1055/s-0029-1242458 21037809 PMC2796096

[B19] Hallas J. Christensen R. Andersen M. Friis S. Bjerrum L. (2012). Long term use of drugs affecting the renin‐angiotensin system and the risk of cancer: a population‐based case‐control study. Br. J. Clin. Pharmacol. 74 (1), 180–188. 10.1111/j.1365-2125.2012.04170.x 22243442 PMC3394143

[B20] Iacopetta B. (2002). Are there two sides to colorectal cancer? Int. J. cancer 101 (5), 403–408. 10.1002/ijc.10635 12216066

[B21] Jansen L. Below J. Chang‐Claude J. Brenner H. Hoffmeister M. (2012). Beta blocker use and colorectal cancer risk: population‐based case‐control study. Cancer 118 (16), 3911–3919. 10.1002/cncr.26727 22585669

[B22] Khalil H. Zeltser R. (2022). Antihypertensive medications. Florida, United States: StatPearls Publishing. 32119466

[B23] Li D. (2018). Recent advances in colorectal cancer screening. Chronic Dis. Transl. Med. 4 (3), 139–147. 10.1016/j.cdtm.2018.08.004 30276360 PMC6160607

[B24] Lin J. S. Piper M. A. Perdue L. A. Rutter C. Webber E. M. O’Connor E. (2016). Screening for colorectal cancer: a systematic review for the US preventive services task force. Available at: https://pubmed.ncbi.nlm.nih.gov/27441328/. 27441328

[B25] Lin O. S. Kozarek R. A. Cha J. M. (2014). Impact of sigmoidoscopy and colonoscopy on colorectal cancer incidence and mortality: an evidence-based review of published prospective and retrospective studies. Intestinal Res. 12 (4), 268–274. 10.5217/ir.2014.12.4.268 PMC4214952 25374491

[B26] Lund J. L. Richardson D. B. Stürmer T. (2015). The active comparator, new user study design in pharmacoepidemiology: historical foundations and contemporary application. Curr. Epidemiol. Rep. 2, 221–228. 10.1007/s40471-015-0053-5 26954351 PMC4778958

[B27] Mackenzie I. S. Morant S. V. Wei L. Thompson A. M. MacDonald T. M. (2017). Spironolactone use and risk of incident cancers: a retrospective, matched cohort study. Br. J. Clin. Pharmacol. 83 (3), 653–663. 10.1111/bcp.13152 27735065 PMC5306481

[B28] Makar G. A. Holmes J. H. Yang Y.-X. (2014). Angiotensin-converting enzyme inhibitor therapy and colorectal cancer risk. J. Natl. Cancer Inst. 106 (2), djt374. 10.1093/jnci/djt374 24431411 PMC3952198

[B29] Mandel J. S. Church T. R. Bond J. H. Ederer F. Geisser M. S. Mongin S. J. (2000). The effect of fecal occult-blood screening on the incidence of colorectal cancer. N. Engl. J. Med. 343 (22), 1603–1607. 10.1056/NEJM200011303432203 11096167

[B30] Mann J. (2020). Choice of drug therapy in primary (essential) hypertension. Available at: https://www.uptodate.com/contents/choice-of-drug-therapy-in-primary-essential-hypertension.

[B31] Murphy R. A. Darvishian M. Qi J. Chen Y. Chu Q. Vena J. (2022). Lifestyle factors and lung cancer risk among never smokers in the Canadian Partnership for Tomorrow’s Health (CanPath). Cancer Causes Control 33 (6), 913–918. 10.1007/s10552-022-01566-x 35302182

[B32] Padwal R. S. Bienek A. McAlister F. A. Campbell N. R. Outcomes Research Task Force of the Canadian Hypertension Education Program (2016). Epidemiology of hypertension in Canada: an update. Can. J. Cardiol. 32 (5), 687–694. 10.1016/j.cjca.2015.07.734 26711315

[B33] Pharmacists A. S. o.H.-S. Service A. H. F. (2002). AHFS drug information. Bethesda: American Society of Health-System Pharmacist.

[B34] Qi J. An R. Bhatti P. Spinelli J. J. Murphy R. A. (2022). Anti-hypertensive medications and risk of colorectal cancer: a systematic review and meta-analysis. Cancer Causes Control 33, 801–812. 10.1007/s10552-022-01570-1 35314908

[B35] Rabi D. M. McBrien K. A. Sapir-Pichhadze R. Nakhla M. Ahmed S. B. Dumanski S. M. (2020). Hypertension Canada’s 2020 comprehensive guidelines for the prevention, diagnosis, risk assessment, and treatment of hypertension in adults and children. Can. J. Cardiol. 36 (5), 596–624. 10.1016/j.cjca.2020.02.086 32389335

[B36] Si H. Yang Q. Hu H. Ding C. Wang H. Lin X. (2021). Colorectal cancer occurrence and treatment based on changes in intestinal flora. Seminars Cancer Biol. 70, 3–10. 10.1016/j.semcancer.2020.05.004 32404293

[B37] Sluggett J. K. Koponen M. Bell J. S. Taipale H. Tanskanen A. Tiihonen J. (2020). Metformin and risk of Alzheimer’s disease among community-dwelling people with diabetes: a national case-control study. J. Clin. Endocrinol. Metabolism 105 (4), dgz234–e972. 10.1210/clinem/dgz234 31778170

[B38] Stricker B. C. Stijnen T. (2010). Analysis of individual drug use as a time-varying determinant of exposure in prospective population-based cohort studies. Eur. J. Epidemiol. 25, 245–251. 10.1007/s10654-010-9451-7 20358262 PMC2850996

[B39] Summers R. M. (2010). Polyp size measurement at CT colonography: what do we know and what do we need to know? Radiology 255 (3), 707–720. 10.1148/radiol.10090877 20501711 PMC2875919

[B40] Tamim H. Monfared A. T. LeLorier J. (2007). Application of lag‐time into exposure definitions to control for protopathic bias. Pharmacoepidemiol. drug Saf. 16 (3), 250–258. 10.1002/pds.1360 17245804

[B41] Team R. C. (2016). R: a language and environment for statistical computing. Vienna, Austria: R Foundation for Statistical Computing.

[B42] Tenenbaum A. Motro M. Jonas M. Fisman E. Z. Grossman E. Boyko V. (2001). Is diuretic therapy associated with an increased risk of colon cancer? Am. J. Med. 110 (2), 143–145. 10.1016/s0002-9343(00)00674-4 11165556

[B43] van der Knaap R. Siemes C. Coebergh J. W. W. van Duijn C. M. Hofman A. Stricker B. H. C. (2008). Renin‐angiotensin system inhibitors, angiotensin I‐converting enzyme gene insertion/deletion polymorphism, and cancer: the Rotterdam Study. Cancer 112 (4), 748–757. 10.1002/cncr.23215 18181094

[B44] Vik S. A. Maxwell C. J. Hogan D. B. (2004). Measurement, correlates, and health outcomes of medication adherence among seniors. Ann. Pharmacother. 38 (2), 303–312. 10.1345/aph.1D252 14742770

[B45] Wang K.-L. Liu C.-J. Chao T.-F. Huang C.-M. Wu C.-H. Chen T.-J. (2013). Long-term use of angiotensin II receptor blockers and risk of cancer: a population-based cohort analysis. Int. J. Cardiol. 167 (5), 2162–2166. 10.1016/j.ijcard.2012.05.096 22709730

[B46] Wiens M. Etminan M. Gill S. Takkouche B. (2006). Effects of antihypertensive drug treatments on fracture outcomes: a meta‐analysis of observational studies. J. Intern. Med. 260 (4), 350–362. 10.1111/j.1365-2796.2006.01695.x 16961672

[B47] Winawer S. O'brien M. Waye J. Kronborg O. Bond J. Frühmorgen P. (1990). Risk and surveillance of individuals with colorectal polyps. Who collaborating centre for the prevention of colorectal cancer. Bull. World Health Organ. 68 (6), 789–795. 2073716 PMC2393163

[B48] Zhang Z. (2016). Missing data imputation: focusing on single imputation. Ann. Transl. Med. 4 (1), 9. 10.3978/j.issn.2305-5839.2015.12.38 26855945 PMC4716933

