# Supplementary material for: Antihypertensive medications and risk of colorectal cancer in British Columbia
Source: Front Pharmacol. 2023 Nov 7;14:1301423. doi: 10.3389/fphar.2023.1301423 (PMC10662292; doi:10.3389/fphar.2023.1301423)
Supplement: Supplementary file 1 [file Table1.DOCX]

| **SUPPLEMENTARY TABLE 1 Classes, subclasses and individual commonly prescribed antihypertensive medications.** | | | | |
| --- | --- | --- | --- | --- |
| Class | Subclass | Individual medication | AHFS code | DDD |
| ACEIs | NA | Ramipril | 392027 | 2.5 mg |
|  |  | Captopril | 382823 | 50 mg |
|  |  | Cilazapril | 395018 | 2.5 mg |
|  |  | Quinapril | 392026 | 15 mg |
|  |  | Trandolapril | 397010 | 2 mg |
|  |  | Benazepril | 392011 | 7.5 mg |
|  |  | Enalapril | 386022 | 10 mg |
|  |  | Fosinopril | 392020 | 15 mg |
|  |  | Lisinopril | 392051 | 10 mg |
|  |  | Perindopril | 399017 | 4 mg |
|  |  |  |  |  |
| ARBs | NA | Candesartan | 302015 | 8 mg |
|  |  | Eprosartan | 302013 | 600 mg |
|  |  | Irbesartan | 398009 | 150 mg |
|  |  | Losartan | 395008 | 50 mg |
|  |  | Olmesartan | 302020 | 20 mg |
|  |  | Telmisartan | 302016 | 40 mg |
|  |  | Valsartan | 312003 | 80 mg |
|  |  |  |  |  |
| BBs | β1 blockers | Atenolol | 384031 | 75 mg |
|  |  | Bisoprolol | 393024 | 10 mg |
|  |  | Metoprolol | 382864 | 150 mg |
|  |  | Acebutolol | 387003 | 400 mg |
|  |  | Betaxolo | 393047 | 200 mg |
|  |  | Esmolol | 387013 | 2500 mg |
|  |  |  |  |  |
|  | β1/β2 blockers | Nadolol | 382666 | 160 mg |
|  |  | Propranolol | 382607 | 160 mg |
|  |  | Timolol | 384029 | 200 mg |
|  |  | Pindolol | 384032 | 15 mg |
|  |  | Labetalol | 385034 | 600 mg |
|  |  | Nebivolol | 308012 | 5 mg |
|  |  | Sotalol | 393010 | 160 mg |
|  |  | Carvedilol | 397042 | 37.5 mg |

| Class | Subclass | Individual medication | AHFS code | DDD |
| --- | --- | --- | --- | --- |
| CCBs | DHPs | Amlodipine | 392044 | 5 mg |
|  |  | Felodipine | 392016 | 5 mg |
|  |  | Nifedipine | 384028 | 30 mg |
|  |  | Nicardipine | 395032 | 90 mg |
|  |  | Nimodipine | 389010 | 300 mg |
|  |  |  |  |  |
|  | NDHPs | Diltiazem | 384027 | 240 mg |
|  |  | Verapamil | 384030 | 240 mg |
| Diuretics | Thiazide diuretics | Chlorthalidone | 382342 | 25 mg |
|  |  | Chlorothiazide | 382341 | 25 mg |
|  |  | HCTZ | 382571 | 25 mg |
|  |  | Indapamide | 384062 | 2.5 mg |
|  |  | Metolazone | 382345 | 5 mg |
|  |  | Bendroflumethiazide | 382573 | 2.5 mg |
|  |  | Methyclothiazide | 382569 | 5 mg |
|  |  | Polythiazide | 382568 | 1 mg |
|  |  |  |  |  |
|  | Loop diuretics | Bumetanide | 384051 | 1 mg |
|  |  | Ethacrynic acid | 382857 | 50 mg |
|  |  | Furosemide | 382858 | 40 mg |
|  |  | Torsemide | 394018 | 15 mg |
|  |  |  |  |  |
|  | Potassium sparing  diuretics | Spironolactone | 382627 | 75 mg |
|  |  | Amiloride | 381046 | 10 mg |
|  |  | Triamterene | 382337 | 100 mg |
|  |  | Eplerenone | 302037 | 50 mg |
| Abbreviations: ACEI, angiotensin-converting enzyme inhibitor; AHFS, American Hospital Formulary Service; ARB, angiotensin II receptor blocker; BB, beta-blocker; CCB, calcium channel blocker; DHP, dihydropyridine; NDHP, non-dihydropyridine; HCTZ, hydrochlorothiazide; DDD, defined daily dose; NA, not applicable | | | | |
