# Supplementary material for: Antihypertensive medications and risk of colorectal cancer in British Columbia
Source: Front Pharmacol. 2023 Nov 7;14:1301423. doi: 10.3389/fphar.2023.1301423 (PMC10662292; doi:10.3389/fphar.2023.1301423)
Supplement: Supplementary file 2 [file Table2.DOCX]

| **SUPPLEMENTARY TABLE 2 Distributions of missingness in covariates.** | | |
| --- | --- | --- |
| Covariates | All  (n= 1,693,297) | BCGP  (n= 19,819) |
| Frequency (%) |  |  |
| Sex | 403 (0.02%) | 0 (0.00%) |
| Income quintile at baseline | 227,481 (13.4%) | 1,811 (9.14%) |
| Health authority at baseline | 202,580 (12.0%) | 1,640 (8.27%) |
| Birth year | 0 (0.00%) | 0 (0.00%) |
| Ethnicity | NA | 644 (3.25%) |
| Education | NA | 128 (0.65%) |
| Marital status | NA | 107 (0.54%) |
| Household income | NA | 1,291 (6.51%) |
| BMI | NA | 0 (0.00%) |
| Family history of all cancers | NA | 567 (2.86%) |
| Sleep duration | NA | 0 (0.00%) |
| Vegetable and fruit consumption | NA | 0 (0.00%) |
| Alcohol consumption | NA | 1,472 (7.43%) |
| Smoking status | NA | 248 (1.25%) |
| Moderate-to-vigorous  physical activity | NA | 1,479 (7.46%) |
| Fecal occult blood test | NA | 1,175 (5.93%) |
| Sigmoidoscopy or colonoscopy | NA | 240 (1.21%) |
| Polyp removal | NA | 566 (2.86%) |
| Abbreviations: BCGP, British Columbia Generations Project; BMI, Body mass index; NA, not applicable | | |
